# Exposure to Hyperchloremia Is Associated with Poor Early Recovery of Kidney Graft Function after Living-Donor Kidney Transplantation: A Propensity Score-Matching Analysis

**DOI:** 10.3390/jcm8070955

**Published:** 2019-07-02

**Authors:** Jin Go, Sun-Cheol Park, Sang-Seob Yun, Jiyeon Ku, Jaesik Park, Jung-Woo Shim, Hyung Mook Lee, Yong-Suk Kim, Young Eun Moon, Sang Hyun Hong, Min Suk Chae

**Affiliations:** 1 Department of Surgery, Division of Vascular and Transplant Surgery, Mediplex Sejong Hospital, Incheon 21080, Korea; 2 Department of Surgery, Division of Vascular and Transplant Surgery, Seoul St. Mary’s Hospital, College of Medicine, The Catholic University of Korea, Seoul 06591, Korea; 3 Department of Anesthesiology and Pain Medicine, Bucheon St. Mary’s Hospital, College of Medicine, The Catholic University of Korea, Seoul 06591, Korea; 4 Department of Anesthesiology and Pain Medicine, Seoul St. Mary’s Hospital, College of Medicine, The Catholic University of Korea, 222, Banpo-daero, Seocho-gu, Seoul 06591, Korea

**Keywords:** hyperchloremia, kidney graft dysfunction, living donor kidney transplantation

## Abstract

The effects of hyperchloremia on kidney grafts have not been investigated in patients undergoing living-donor kidney transplantation (LDKT). In this study, data from 200 adult patients undergoing elective LDKT between January 2016 and December 2017 were analyzed after propensity score (PS) matching. The patients were allocated to hyperchloremia and non-hyperchloremia groups according to the occurrence of hyperchloremia (i.e., ≥110 mEq/L) immediately after surgery. Poor early graft recovery was defined as estimated glomerular filtration rate (eGFR) < 60 mL/min/1.73 m^2^ during the first 48 hours after surgery. After PS matching, no significant differences in perioperative recipient or donor graft parameters were observed between groups. Although the total amount of crystalloid fluid infused during surgery did not differ between groups, the proportions of main crystalloid fluid type used (i.e., 0.9% normal saline vs. Plasma Solution-A) did. The eGFR increased gradually during postoperative day (POD) 2 in both groups. However, the proportion of patients with eGFR > 60 mL/min/1.73 m^2^ on POD 2 was higher in the non-hyperchloremia group than in the hyperchloremia group. In this PS-adjusted analysis, hyperchloremia was significantly associated with poor graft recovery on POD 2. In conclusion, exposure to hyperchloremia may have a negative impact on early graft recovery in LDKT.

## 1. Introduction

Acute kidney injury (AKI) is a common complication that increases the risk of poor graft outcome in patients undergoing kidney transplantation (KT). Kidney grafts seem to be vulnerable to poor early function recovery because of various types of acute perioperative damage, such as ischemia-reperfusion injury, immunological insult, medication toxicity, and surgical stress [1]. In living-donor kidney transplantation (LDKT), better graft quality and patient condition result in better early graft function compared with deceased-donor KT. However, the reported incidence of poor early graft function after LDKT is 10–20%, and this early graft dysfunction is closely related closely to an increased risk of long-term graft failure [2,3]. 

Chloride is a major anion in extracellular fluid and an essential element for plasma tonicity. Chloride plays a role in maintaining aspects of homeostasis, such as acid balance, muscular activation, and immunological response [4]. Hyperchloremia is a potential risk factor for AKI in critically ill patients admitted to the intensive care unit (ICU) [5,6,7,8]. In patients undergoing noncardiac surgery, the perioperative development of hyperchloremia was reported to be independently associated with morbidity and mortality risks [9]. These harmful effects of hyperchloremia have largely been investigated in the context of chloride-rich solution resuscitation, which has been found to have a detrimental impact on kidney function [10,11]. However, the relationship between hyperchloremia and AKI has been investigated only in native (i.e., not transplanted) kidneys, in the context of clinical conditions such as sepsis, subarachnoid hemorrhage, and abdominal surgery [5,6,7,8,12].

To date, the effects of hyperchloremia on kidney grafts have not been investigated in patients undergoing LDKT. We investigated the association between hyperchloremia and early graft function recovery. In addition, postoperative outcomes, such as the requirement for renal replacement therapy (RRT), graft rejection, and mortality, were investigated according to the occurrence of hyperchloremia.

## 2. Patients and Methods

### 2.1. Ethical Considerations

This study was approved by the Institutional Review Board and Ethics Committee of Seoul St. Mary’s Hospital (KC19RESI0088) and was performed in accordance with the Declaration of Helsinki. The requirement for informed consent was waived due to the retrospective study design.

### 2.2. Study Population

The study population consisted of 330 adult patients (i.e., age ≥ 19 years) who underwent elective LDKT at Seoul St. Mary’s Hospital between January 2016 and December 2017. Pediatric patients (i.e., age < 19 years), those undergoing deceased-donor or ABO-incompatible KT, patients undergoing multiorgan transplantation including the kidney, and those undergoing re-transplantation were excluded from the study because these patients require various and complex immunosuppression regimens or surgical technique application [13,14,15,16,17]. Patients with defective or missing recipient and donor graft data were also excluded. Based on the exclusion criteria, 29 patients were not included in the study. In total, 301 patients were initially enrolled and their data were included in propensity score (PS)-matching analysis; data from 200 matched patients were included in the final analysis.

### 2.3. Living Donor Kidney Transplantation

The surgical technique for LDKT involved an initial hockey-stick (i.e., pararectal inverted J-shaped curvilinear) incision and exposure of the right pelvic fossa. After back table preparation of the graft, end-to-side anastomoses between the recipient external iliac artery/vein and the graft renal artery/vein were performed using Prolene 6.0 resorbable monofilament (Ethicon, Somerville, NJ, USA). Subsequently, ureteroneocystostomy was performed with insertion of a double-J stent (INLAY ureteral stent; Bard Medical, Covington, GA, USA) using the Lich-Grègoir technique [18,19]. After careful hemostasis and re-assessment of the vascular anastomosis and renal pedicle area, closed drains were placed and the wound was closed.

Balanced anesthesia was applied using 1–2 mg/kg propofol (Fresenius Kabi, Bad Homburg, Germany) and 0.6 mg/kg rocuronium (Merck Sharp & Dohme Corp., Kenilworth, NJ, USA), and maintained using 2.0–6.0% desflurane (Baxter, Deerfield, IL, USA) with medical air/oxygen and continuous remifentanil (Hanlim Pharmaceutical Co., Ltd., Seoul, Republic of Korea) infusion at a rate of 0.1–0.5 μg/kg/min, as appropriate. The maintenance of appropriate hypnotic depth between 40 and 50 was ensured with a Bispectral Index™ instrument (Medtronic, Minneapolis, MN, USA). Central venous pressure (CVP) was monitored using a central venous catheter (Arrow, Morrisville, NC, USA) inserted before surgery. The optimal hemodynamic status was adjusted to a mean arterial pressure of ≥65 mmHg with infusion of dopamine (Reyon Pharm. Co., Ltd., Seoul, Republic of Korea) at a rate of 5–10 μg/kg/min. Mannitol (Daihan Pharm. Co., Ltd., Seoul, Republic of Korea) was used at doses of 20–50 g to promote urine flow [20]. However, we did not regularly cannulate and/or puncture a radial artery for continuous monitoring of blood pressure or arterial blood gas analysis (ABGA), to avoid arterial injury. We only measured ABGA when oxygen saturation was below 90% using pulse oximetry.

For intraoperative fluid therapy, an isotonic crystalloid fluid (0.9% normal saline (Daihan Pharm Co., Ltd.) or Plasma Solution-A (CJ Healthcare, Seoul, Republic of Korea)) was selected at the discretion of the attending anesthesiologist. The 0.9% normal saline (i.e., chloride-liberal fluid) included sodium chloride (9 g/L; 154 mEq/L sodium; and 154 mEq/L chloride). Plasma Solution-A (i.e., chloride-restrictive fluid) included magnesium chloride (0.3 g/L; 0.37 g/L potassium chloride, 3.68 g/L sodium acetate, 5.26 g/L sodium chloride, and 5.02 g/L sodium gluconate (140 mEq/L sodium, 98 mEq/L chloride, 5 mEq/L potassium, and 3 mEq/L magnesium)). Baseline isotonic crystalloid infusion was based on the estimated fluid maintenance requirements, calculated from the patient’s weight and anticipated tissue trauma [21]. Additional fluid boluses were administered to reach a target CVP of 10–15 mmHg or hydration volume of 50–100 mL/kg, to ensure sufficient flow for the maintenance of adequate kidney graft perfusion and to replace the amount of urine output after graft reperfusion [22]. 

In the immunosuppressive regimen (Table 1), the induction drug was based on interleukin-2 receptor antagonist (i.e., Basiliximab) and T lymphocyte-depleting rabbit-derived anti-thymocyte globulin (i.e., thymoglobulin), and drug maintenance was based on a calcineurin inhibitor (i.e., tacrolimus), mycophenolate mofetil, and steroids. Steroid pulse therapy and/or thymoglobulin rescue therapy were applied in cases of graft rejection. 

### 2.4. Measurement of Serum Chloride Levels

The baseline chloride level was estimated between the day after dialysis and the preoperative period, and serial chloride levels were measured 1 day before surgery, immediately after surgery, and on postoperative days (PODs) 1 and 2. The chloride level was analyzed by indirect potentiometry (Clinical Analyzer 7600; Hitachi, Tokyo, Japan). 

The patients were divided according to the presence or absence of hyperchloremia (defined as ≥110 mEq/L [6,23]) immediately after surgery into the hyperchloremia group and the non-hyperchloremia group, respectively.

### 2.5. Definition of Poor Early Recovery of Kidney Graft Function

Kidney graft function was quantified based on the estimated glomerular filtration rate (eGFR), calculated using the Modification of Diet in Renal Disease formula: eGFR = 175 × standardized serum chloride^−1.154^ × age^−0.203^ × 1.212 (if black) × 0.742 (if female) [24]. The baseline eGFR was estimated between the day after dialysis and the preoperative period, and serial eGFRs were measured immediately after surgery and on PODs 1 and 2. Based on the eGFR, the degree of graft function was classified as chronic kidney disease (CKD) stage I (i.e., normal kidney function and eGFR ≥ 90 mL/min/1.73 m^2^), stage II (i.e., mild loss of kidney function and eGFR 60–89 mL/min/1.73 m^2^), stage IIIa (i.e., mild to moderate loss of kidney function and eGFR 45–59 mL/min/1.73 m^2^), stage IIIb (i.e., moderate to severe loss of kidney function and eGFR 30–44 mL/min/1.73 m^2^), stage IV (i.e., severe loss of kidney function and eGFR 15–29 mL/min/1.73 m^2^), or stage V (i.e., kidney failure and eGFR < 15 mL/min/1.73 m^2^) [25]. 

In the present study, poor early recovery of kidney graft function, defined as eGFR < 60 mL/min/1.73 m^2^ during the first 48 hours after surgery [26], was the primary outcome. 

### 2.6. Clinical Variables

Preoperative and intraoperative recipient and donor graft factors in the non-hyperchloremia and hyperchloremia groups were assessed by PS matching analysis. Preoperative recipient factors included age, sex, body mass index (BMI), comorbidities (i.e., diabetes mellitus (DM) and hypertension), dialysis history and duration, and laboratory variables (i.e., white blood cell count, platelet count, and hemoglobin, sodium, chloride, potassium, albumin, creatinine, and glucose concentrations). Intraoperative factors included the operation time, averages of vital signs (i.e., systolic and diastolic blood pressures, heart rate, CVP, and body temperature), and total amounts of crystalloid infusion, urine output, and blood loss. Donor graft factors included age, sex, BMI, graft weight, total graft ischemic time, and human leukocyte antigen level. 

Postoperative clinical factors included RRT requirement, biopsy-proven graft rejection [27], and patient mortality during the follow-up period.

### 2.7. Statistical Analysis

The normality of continuous data was assessed using the Shapiro-Wilk test. Continuous data are expressed as medians and interquartile ranges, and categorical data are expressed as numbers and proportions. PS matching analysis was applied to reduce the impact of potential confounding factors on intergroup differences based on hyperchloremia [28,29]. PSs were derived to match patients at a 1:1 ratio using greedy matching algorithms without replacement. Perioperative recipient and donor graft factors were compared using the Mann–Whitney *U* test and *χ*^2^ test or Fisher’s exact test, as appropriate. Wilcoxon’s signed-rank sum test and McNemar’s test were used for analysis of the pair-matched data. Postoperative changes in the proportions of patients with kidney graft function classified as eGFR ≥ 60 vs. 30–59 vs. < 30 mL/min/1.73 m^2^ were analyzed using Cochran’s *Q* test with the McNemar post hoc test. The association of hyperchloremia with poor early recovery of kidney graft function was evaluated by multivariable logistic regression analysis with PS adjustment. The values are presented as odds ratios with 95% confidence intervals. All tests were two sided, and *p* < 0.05 was taken to indicate statistical significance. All statistical analyses were performed using R software version 2.10.1 (R Foundation for Statistical Computing, Vienna, Austria) and SPSS for Windows (ver. 24.0; SPSS Inc., Chicago, IL, USA).

## 3. Results

### 3.1. Demographic Characteristics of Patients Undergoing LDKT

The total study population of 301 patients comprised 188 (62.5%) males and 113 (35.5%) females with an average age of 49 ± 11 years and average BMI of 23.1 ± 3.5 kg/m^2^. The incidences of DM and hypertension were 28.6% (*n* = 86) and 58.1% (*n* = 175), respectively. Dialysis was performed in 220 (73.1%) patients for an average duration of 30 ± 53 months. The average eGFR was 7.6 ± 3.5 mL/min/1.73 m^2^. A total of 295 (98.0%) patients had CKD stage V (i.e., eGFR <15 mL/min/1.73 m^2^), five (1.7%) patients had CKD stage IV (i.e., eGFR 15–29 mL/min/1.73 m^2^), and one (0.03%) patient had CKD stage IIIb (i.e., eGFR 30–44 mL/min/1.73 m^2^).

### 3.2. Comparison of Perioperative Factors before and after PS Matching

Before PS matching, there were significant differences between groups in preoperative findings (i.e., dialysis duration, sodium and glucose levels), intraoperative findings (i.e., average diastolic blood pressure and total amount of hemorrhage), and donor graft parameters (i.e., total graft ischemic time; Table 2). After PS matching, there were no significant differences in perioperative recipient or donor graft parameters between groups.

### 3.3. Comparison of Main Crystalloid Fluid Infusion during Surgery and Electrolyte Values Immediately after Surgery in PS-Matched Patients

The total amount of crystalloid fluid infused during surgery did not differ between groups. However, the proportions of main crystalloid fluid type used (i.e., 0.9% normal saline vs. Plasma Solution-A) differed between the groups (Table 3). With regard to electrolyte values immediately after surgery, serum sodium and potassium levels were similar in the two groups, but the serum chloride level was higher and the change in chloride level was greater in the hyperchloremia group.

### 3.4. Serial Changes in eGFR until POD 2 in PS-Matched Patients

The eGFR, as an indicator of kidney graft function, increased gradually until POD 2 in both groups (Table 4). However, the proportion of patients with eGFR > 60 mL/min/1.73 m^2^ increased significantly from immediately after surgery to PODs 1 and 2 in the non-hyperchloremia group (Table 5). On POD 2, the proportion of patients with eGFR > 60 mL/min/1.73 m^2^ was larger and the proportion of patients with eGFR < 30 mL/min/1.73 m^2^ was smaller in the non-hyperchloremia group than in the hyperchloremia group.

### 3.5. Association of Hyperchloremia with Kidney Graft Function (i.e., eGFR ≤ 60 mL/min/1.73 m^2^) on POD 2 

Hyperchloremia was associated with poor graft recovery on POD 2 in the whole study population and in PS-matched patients (Table 6). After PS adjustment, hyperchloremia remained an independent factor related to poor graft recovery.

### 3.6. Postoperative Clinical Outcomes in PS-Matched Patients

In the hyperchloremia group, five (5.0%) patients required RRT due to poor graft function, five (5.0%) patients suffered graft rejection, and two (2.0%) patients died. In the non-hyperchloremia group, two (2.0%) patients required RRT, one (1.0%) patient suffered graft rejection, and one (1.0%) patient died. These postoperative outcomes did not differ significantly between groups. 

## 4. Discussion

The main finding of our study was that hyperchloremia was associated with poor early recovery of graft function after LDKT in an analysis adjusted for clinical factors related to kidney graft function by PS matching. Patients without hyperchloremia showed appropriate graft function recovery during POD 2, as indicated by the increase in proportion of patients with eGFR > 60 mL/min/1.73 m^2^ and decrease in the proportion of patients with eGFR < 30 mL/min/1.73 m^2^. The proportion of patients with adequate graft function (i.e., eGFR > 60 mL/min/1.73 m^2^) on POD 2 was larger in the non-hyperchloremia group than in the hyperchloremia group.

Many studies have examined the impact of hyperchloremia on AKI in critically ill patients, but debate persists regarding the relation between hyperchloremia and the occurrence of AKI [5,6,23,30,31]. A study of severely septic patients in the ICU showed that hyperchloremia was common in these patients due to aggressive fluid resuscitation, but that neither hyperchloremia nor an increase in the serum chloride level was associated with an increased risk of AKI development within the first three days of ICU admission [23]. In patients undergoing craniotomy for intracranial hemorrhage, hyperchloremia due to infusion of 0.9% NaCl solution was related to a decrease in the water shift across the blood–brain barrier, leading to metabolic acidosis, but did not directly cause AKI [30,32]. However, in another study of patients undergoing craniotomy for primary brain tumor resection, the occurrence of hyperchloremia within 3 PODs appeared to aggravate early kidney function [33]. In a study of patients with subarachnoid hemorrhage in the neurocritical care unit, those who developed AKI had a higher average serum chloride level than did those without AKI, despite similar chloride loading [5]. Suetrong et al. [6] suggested that the maximum serum chloride level (i.e., ≥ 110 mmol/L) and an increase ≥ 5 mmol/L within 48 hours after ICU admission due to severe sepsis or septic shock were the predominant factors associated with the development of AKI. Another study conducted by Marouli et al. [12] suggested that a high intraoperative chloride load (i.e., > 500 mEq) played a significant role in the development of AKI within 48 hours after major abdominal surgery. In a prospective ICU study, a lesser intravenous chloride supply was associated with significant reduction in the worst stages of AKI and the requirement for RRT. In fluid management, the infusion of a chloride-restrictive fluid (i.e., Plasma-Lyte 148), rather than a chloride-liberal fluid (i.e., 0.9% normal saline), may effectively attenuate the increase in serum creatinine level from baseline to peak during the ICU stay [34]. 

Patients with chronic kidney dysfunction and those undergoing RRT have been routinely excluded from many studies of AKI because the initial kidney status is one of the most critical factors contributing to the development of AKI after surgery or ICU admission [35]. In contrast, our study included patients with end-stage renal disease, most of whom were receiving dialysis, with kidney grafts rendered vulnerable by ischemic injury [1]. The impact of hyperchloremia on graft function recovery in the early period has not been investigated fully in such LDKT settings. As various preoperative and intraoperative risk factors may be related to postoperative graft function, these clinical risk factors were matched between patients with and without hyperchloremia to reduce selection bias using a PS-based method [29,36]. Therefore, the results of the present study suggest that hyperchloremia is an independent risk factor for inappropriate graft recovery after LDKT. The intraoperative infusion of a large amount of 0.9% normal saline may be related to an increase in the chloride load and consequent effects on kidney grafts because 0.9% saline contains 50% more chloride than serum (154 vs. 100 mEq/L) [37]. Postoperatively, however, the sodium and potassium levels did not differ between our study groups, suggesting that they are not related to an increased risk of AKI development, consistent with previous findings [5,6]. In animal experiments [38,39], a potential explanation for chloride-load kidney injury was suggested to be the dysregulation of tubuloglomerular feedback caused by chloride reaching the macula densa, which caused renal afferent arteriole vasoconstriction related to decreased renal cortical tissue perfusion and the development of tissue ischemia. A second explanation was proposed to involve renal interstitial edema related to fluid overload resulting in intracapsular hypertension or vasomotor nephropathy. Although the specific pathophysiology of the relationship between hyperchloremia and graft dysfunction in patients undergoing LDKT remains unclear, exposure of the kidney graft to high serum chloride levels may have a negative effect on functional recovery and prolong the recovery period.

This study had some limitations. First, although confounding factors were adjusted between patients with and without hyperchloremia by PS matching, hidden biases attributable to unknown factors could not be completely excluded. Second, we were not able to determine the amounts of chloride infused from the patients’ records, so the analysis was based on the serum chloride concentrations. Third, our observations did not elucidate the pathophysiology underlying the relationship between hyperchloremia and kidney graft function recovery. Fourth, because of the possibility of graft failure requiring RRT, such as arteriovenous fistula [40], we did not routinely perform arterial procedures, such as ABGA. We were unable to determine the effects of metabolic acidosis on early graft recovery. Finally, the power to identify associations of hyperchloremia with the requirement for RRT, rejection, and mortality was limited because the sample was small.

## 5. Conclusions

Exposure to hyperchloremia may have a negative effect on the early recovery of kidney grafts injured by ischemia in LDKT. The infusion of large amounts of chloride-rich fluid seems to be a major factor contributing to increased serum chloride levels, and this chloride load may play a role in prolonging kidney graft recovery in the early postoperative period. Previous studies have shown that chloride loads cause renal vasoconstriction and interstitial edema, thereby decreasing renal blood flow and perfusion, and consequently reducing the GFR and urine output [41,42]. However, the potential pathophysiological relationships in the KT-specific setting have not been clarified. Therefore, further studies are required to determine the association between the chloride load and transplanted kidney graft functional recovery.

## Figures and Tables

**Table 1:** Immunosuppressive regimen in living donor kidney transplantation.

Maintain Immune Therapy			
**Induction Therapy**	**Basiliximab**	**20 mg IV. at Surgical Day and POD 4**	
	Thymoglobulin	1.25 mg/kg IV at surgical day and during POD 4	
Maintenance therapy	Tacrolimus	0.06 mg/kg on 2 day before surgery 0.05 mg/kg on 1 day before surgery	Dosage control under serum level after surgery
	Mycophenolate	MMF 750 mg/MYF 540 mg p.o. q.d. and raise bid after surgery	Under 50 kg patient MMF 500 mg/MYF 360 mg p.o. bid
	Steroids	Prednisone 125 mg IV qid and reduce until 60 mg tid	p.o. change until 10 mg p.o. bid for prednisolone
**Immune Therapy for Graft Rejection**			
Steroid pulse therapy		Prednisone 500 mg IV/day	Cessation of IV within 5 days and switch to p.o. medication
Thymoglobulin rescue therapy		1.25 mg/kg IV for 5 days	

Abbreviations: POD, postoperative day; MMF; mycophenolate mofetil, MYF; mycophenolate sodium, IV; intravenous, p.o.; per oral; q.d., quaque die (every day); bid, bis in die (twice a day); qid, quarter in die (four times a day).

**Table 2:** Comparison of clinical perioperative factors between the non-hyperchloremia and hyperchloremia groups before and after propensity score matching analysis.

	Before PS Matching				After PS Matching			
Group	No Hyperchloremia	Hyperchloremia	*p*	SD	No Hyperchloremia	Hyperchloremia	*p*	SD
n	201	100			100	100		
**Recipient parameter**								
**Preoperative finding**								
Age (years)	50 (42–56)	53 (42–58)	0.087	0.118	52 (46–59)	53 (42–58)	0.850	−0.040
Sex (male)	121 (60.2%)	67 (67.0%)	0.251	−0.144	68 (68.0%)	67 (67.0%)	0.880	0.021
Body mass index (kg/m^2^)	22.7 (20.7–25.2)	23.1 (21.2–25.2)	0.398	0.131	23.2 (21.1–25.6)	23.1 (21.2–25.2)	0.910	0.040
Comorbidity								
Diabetes mellitus	60 (29.9%)	26 (26.0%)	0.486	−0.087	29 (29.0%)	26 (26.0%)	0.635	−0.068
Hypertension	123 (61.2%)	52 (52.0%)	0.128	−0.183	53 (53.0%)	52 (52.0%)	0.887	−0.020
Dialysis history	141 (70.1%)	79 (79.0%)	0.103	0.216	76 (76.0%)	79 (79.0%)	0.611	0.073
Dialysis duration (month)	2.0 (0.0–30.0)	6.0 (1.0–57.0)	0.008	0.238	3.0 (0.0–37.5)	6.0 (1.0–57.0)	0.171	0.170
Laboratory analysis								
WBC count (x 10^9^/L)	6.7 (5.1–8.8)	6.3 (4.7–8.1)	0.090	−0.238	6.6 (4.8–7.7)	6.3 (4.7–8.1)	0.581	−0.081
Hemoglobin (g/dL)	10.7 (9.5–12.0)	10.9 (9.9–11.9)	0.333	0.145	10.7 (9.5–12.0)	10.9 (9.9–11.9)	0.433	0.121
Platelet count (x 10^9^/L)	178.0 (146.0–225.5)	183.0 (139.0–231.0)	0.874	−0.009	181.0 (149.0–236.8)	183.0 (139.0–231.0)	0.815	−0.016
Sodium (mEq/L)	137.0 (135.0–139.0)	138.0 (135.3–139.0)	0.018	0.175	137.0 (135.0–139.0)	138.0 (135.3–139.0)	0.094	0.127
Chloride (mEq/L)	98.0 (95.0–101.0)	98.0 (95.0–102.0)	0.387	0.173	98.0 (95.0–101.0)	98.0 (95.0–102.0)	0.698	0.115
Potassium (mEq/L)	4.8 (4.2–5.4)	4.7 (4.2–5.2)	0.400	−0.037	4.8 (4.2–5.4)	4.7 (4.2–5.2)	0.534	0.060
Albumin (g/dL)	4.0 (3.6–4.3)	4.0 (3.7–4.2)	0.553	0.106	3.9 (3.6–4.3)	4.0 (3.7–4.2)	0.383	0.087
Creatinine (mg/dL)	7.8 (6.2–9.9)	7.7 (6.3–10.4)	0.715	0.064	7.7 (6.2–9.5)	7.7 (6.3–10.4)	0.737	0.044
Glucose (mg/dL)	152.0 (126.5–189.0)	140.0 (100.8–171.3)	0.011	0.069	149.0 (121.5–190.8)	140.0 (100.8–171.3)	0.050	−0.034
**Intraoperative finding**								
Surgery time (min)	260 (222–295)	275 (230–309)	0.085	0.195	265 (235–302)	275 (230–309)	0.735	0.001
Average of vital sign								
SBP (mmHg)	125 (117–134)	123 (114–132)	0.186	−0.149	125 (115–131)	123 (114–132)	0.870	0.011
DBP (mmHg)	73 (65–79)	69 (63–77)	0.029	−0.274	71 (63–77)	69 (63–77)	0.525	−0.061
Heart rate (beats/min)	81 (73–89)	80 (71–86)	0.372	−0.039	80 (71–88)	80 (71–86)	0.855	0.012
CVP (mmHg)	10 (8–12)	10 (8–12)	0.174	0.186	10 (8–12)	10 (8–12)	0.991	0.026
Body temperature (℃)	36.3 (36.1–36.5)	36.3 (36.0–36.5)	0.482	−0.067	36.3 (36.1–36.5)	36.3 (36.0–36.5)	0.660	−0.030
Total crystalloid infusion (mL)	2900 (2200–3600)	3100 (2500–4100)	0.066	0.221	3190 (2500–3838)	3100 (2500–4100)	0.990	−0.014
Urine output (mL)	400 (200–700)	350 (100–700)	0.180	−0.131	375 (193–626)	350 (100–700)	0.777	−0.032
Blood loss (mL)	150 (100–250)	200 (150–300)	0.004	0.221	200 (100–300)	200 (150–300)	0.096	0.075
**Donor-graft parameter**								
Age (years)	47 (35–54)	50 (36–56)	0.233	0.111	47 (34–53)	50 (36–56)	0.157	0.162
Sex (male)	103 (51.2%)	47 (47.0%)	0.488	0.085	47 (47.0%)	47 (47.0%)	1.000	0.000
Body mass index (kg/m^2^)	23.7 (21.6–25.9)	23.1 (21.8–25.1)	0.276	−0.197	23.3 (21.0–25.4)	23.1 (21.8–25.1)	0.664	−0.006
Graft weight (g)	184.0 (160.0–216.0)	180.0 (160.5–212.0)	0.398	−0.172	178.0 (158.0–203.5)	180.0 (160.5–212.0)	0.374	0.090
Total graft ischemic time (min)	58 (43–85)	68 (51–126)	0.006	0.265	64 (45–109)	68 (51–126)	0.200	0.113
Human leukocyte antigen analysis								
PRA (positive)								
Class I	63 (31.3%)	36 (36.0%)	0.418	0.097	32 (32.0%)	36 (36.0%)	0.550	0.083
Class II	44 (21.9%)	31 (31.0%)	0.085	0.196	29 (29.0%)	31 (31.0%)	0.758	0.043
DSA (positive)								
Class I	41 (20.4%)	17 (17.0%)	0.481	−0.090	17 (17.0%)	17 (17.0%)	1.000	0.000
Class II	34 (16.9%)	24 (24.0%)	0.142	0.165	23 (23.0%)	24 (24.0%)	0.868	0.023
FCXM (positive)								
T-cell	2 (1.0%)	1 (1.0%)	1.000	0.000	1 (1.0%)	1 (1.0%)	1.000	0.000
B-cell	36 (17.9%)	20 (20.0%)	0.661	0.052	20 (20.0%)	20 (20.0%)	1.000	0.000

Abbreviations: WBC, white blood cell; SBP, systolic blood pressure; DBP, diastolic blood pressure; CVP, central venous pressure; PRA, panel reactive antibody; DSA, donor-specific antibody; FCXM, flow cytometric cross-match. Note: Values are expressed as the median (interquartile range) and number (proportion).

**Table 3:** Comparison of main crystalloid fluid during surgery and electrolyte values immediately after surgery between propensity score-matched non-hyperchloremia and hyperchloremia groups.

Group	No Hyperchloremia	Hyperchloremia	*p*
n	100	100	
Total crystalloid fluid infusion (mL)	3190 (2500–3838)	3100 (2500–4100)	0.990
0.9% normal saline (%)	2.1 (1.7–2.6)	97.7 (95.8–98.4)	<0.001
Plasma Solution-A (%)	97.8 (96.8–98.2)	2.2 (1.6–3.9)	<0.001
Electrolyte values			
Chloride (mEq/L)	107.0 (103.0–108.8)	112.0 (111.0–114.0)	<0.001
^‡^△[Cl^−^] (mEq/L)	8.0 (2.0–12.0)	15.0 (11.0–17.8)	<0.001
Sodium (mEq/L)	140.0 (137.0–142.0)	140.0 (138.0–142.0)	0.783
Potassium (mEq/L)	4.3 (4.0–4.6)	4.4 (4.0–4.9)	0.124

^‡^△[Cl^−^] is defined as the difference in serum chloride between preoperative day and immediately after surgery. Note: Values are expressed as the median and interquartile range.

**Table 4:** Serial changes in estimated glomerular filtration rate during postoperative day 2 between propensity score-matched non-hyperchloremia and hyperchloremia groups.

Group	No Hyperchloremia	Hyperchloremia	*p*
n	100	100	
**eGFR (mL/min/1.73 m^2^)**			
Immediately after surgery	21.5 (9.3–34.3)	17.5 (8.1–29.6)	0.151
Postoperative day 1	54.3 (23.2–77.3)^†††^	43.2 (13.5–70.4)^†††^	0.134
Postoperative day 2	66.7 (33.8–85.7)^†††,§§§^	53.4 (21.3–71.7)^†††,§§§^	0.058

Abbreviation: eGFR, estimated glomerular filtration rate; ^†††^*p* < 0.001 compared to the level immediately after surgery in each group; ^§§§^*p* < 0.001 compared to the level on postoperative day 1 in each group. Note: Values are expressed as the median and interquartile range.

**Table 5:** Comparison of kidney graft function according to estimated glomerular filtration rate during postoperative day 2 between propensity score-matched non-hyperchloremia and hyperchloremia groups.

Group	No Hyperchloremia	Hyperchloremia	*p*
n	100	100	
**eGFR ≥ 60 mL/min/1.73 m^2^**			
Immediately after surgery	6 (6.0%)	2 (2.0%)	0.279
Postoperative day 1	44 (44.0%)^†††^	30 (30.0%)^†††^	0.040
Postoperative day 2	58 (58.0%)^†††,§§§^	38 (38.0%)^†††^	0.005
**eGFR 59–30 mL/min/1.73 m** ^2^			
Immediately after surgery	25 (25.0%)	23 (23.0%)	0.741
Postoperative day 1	28 (28.0%)	32 (32.0%)	0.537
Postoperative day 2	23 (23.0%)	30 (30.0%)	0.262
**eGFR < 30 mL/min/1.73 m^2^**			
Immediately after surgery	69 (69.0%)	75 (75.0%)	0.345
Postoperative day 1	28 (28.0%)^†††^	38 (38.0%)^†††^	0.133
Postoperative day 2	19 (19.0%)^†††,§§^	32 (32.0%)^†††^	0.035

Abbreviation: eGFR, estimated glomerular filtration rate; ^†††^*p* < 0.001 compared to the level immediately after surgery in each group, ^§§^*p* < 0.01 compared to the level on postoperative day 1 in each group, ^§§§^*p* < 0.001 compared to the level on postoperative day 1 in each group. Note: Values are expressed as number and proportion.

**Table 6:** Association of hyperchloremia with poor early graft function (eGFR < 60 ml/min/1.73 m^2^) on postoperative day 2 in living donor kidney transplantation.

	Multivariable Logistic Regression Analysis			
	*ß*	Odds ratio	95% CI	*p*
**In the whole patients (n = 301)**				
Hyperchloremia adjusted for PS	0.592	1.808	1.053–3.104	0.032
**In the PS-matched patients (n = 200)**				
Hyperchloremia adjusted for PS	0.721	2.057	1.146–3.694	0.016

Abbreviation: CI, confidence interval; PS, propensity score.

## Abbreviations

AKI	acute kidney injury
KT	kidney transplantation
LDKT	living donor kidney transplantation
ICU	intensive care unit
RRT	renal replacement therapy
CVP	central venous pressure
POD	postoperative day
eGFR	estimated glomerular filtration rate
MDRD	Modification of Diet in Renal Disease
BMI	body mass index
PS	propensity score
